# Knowledge, Attitude, and Practices Regarding Dengue Fever among Pediatric and Adult In-Patients in Metro Manila, Philippines

**DOI:** 10.3390/ijerph16234705

**Published:** 2019-11-26

**Authors:** Von Ralph Dane M. Herbuela, Ferdinand S. de Guzman, Girly D. Sobrepeña, Andrew Benedict F. Claudio, Angelica Cecilia V. Tomas, Carmina M. Arriola-delos Reyes, Rachele A. Regalado, Mariama M. Teodoro, Kozo Watanabe

**Affiliations:** 1 Graduate School of Science and Engineering, Ehime University, Bunkyo-cho 3, Matsuyama, Ehime 790-8577, Japan; herbuelavonralphdane@gmail.com; 2 Department of Family and Community Medicine, Infectious Diseases and Tropical Medicine, San Lazaro Hospital, Manila 1003, Philippines; fdgzm06@gmail.com; 3 Pediatrics Department, Quezon City General Hospital, Quezon City 1106, Philippines; sobrepenagirly@yahoo.com; 4 Office of Nursing Service, Pasay City General Hospital, Pasay City 1300, Philippines; tomriddle_07@yahoo.com; 5 Department of Pediatrics, College of Medicine, University of the Philippines, Manila 1000, Philippines; anggetomas@gmail.com (A.C.V.T.); minettedr@yahoo.com (C.M.A.-d.R.); 6 Guidance Department, University of Santo Tomas–Angelicum College, Quezon City 1114, Philippines; rachele.arce@angelicum.edu.ph; 7 Counseling and Educational Psychology Department, De La Salle University, Manila 1004, Philippines; mariamateodoro@gmail.com; 8 Biological Control Research Unit, Center for Natural Science and Environmental Research, De La Salle University, Manila 1004, Philippines

**Keywords:** dengue fever, knowledge, attitude, practice, KAP

## Abstract

Background: Knowledge, attitude, and practice (KAP) of in-patients with dengue fever (DF) through hospital-based surveillance has not been done. This study aimed to assess and compare the KAP, identify its predictors, correlation, and protective factors among pediatric and adult patients with DF and community-based controls to structure proactive community-wide DF prevention and control programs. Methods: This case-control study involved clinically or serologically confirmed patients (pediatrics *n* = 233; adults *n* = 17) with DF admitted in three public hospitals and community-based controls in Metro Manila, Philippines. A pretested structured KAP questionnaire was administered to participants to assess their KAP. Results: Pediatric and adult patients had significantly lower mean scores in the practice (*p* < 0.001) domain compared with the pediatric and adult controls. Being in senior high school, having had days in hospital, and rash were predictors of KAP among pediatric patients. Knowledge and attitude of patients with DF did not correlate with their practices against DF. Use of mosquito-eating fish, screen windows, and dengue vaccine were protective factors against DF. Conclusion: The study highlights the importance of behavioral change for knowledge and attitude to have significant effect to practices against DF. Thus, we recommend two comprehensive health programs, Communication for Behavioral Impact (COMBI) and Health Belief Model (HBM).

## 1. Introduction

To date, there is no known cure for dengue fever (DF), the world’s fastest spreading mosquito-borne viral disease transmitted mainly by female *Aedes aegypti* mosquitoes. It causes approximately 390 million cases per year and puts an estimated 3.9 billion people at risk in 128 countries [1,2,3]. DF was first recognized during a dengue epidemic in the Philippines in 1950, and ever since, it has been a substantial and major public health burden causing hospitalization and deaths among children and adults in all regions of the country [2]. The Department of Health (DOH) reported that from 2010 to 2014, there has been an average 170,503 symptomatic cases (178 per 10,000 population) and 750 deaths (0.44% fatality rate) in the Philippines [4].

Metropolitan (Metro) Manila, also known as the National Capital Region (NCR), located in the southwestern part of Luzon, is the capital region of the Philippines [5]. In 2015, according to the DF disease surveillance report of the DOH, Metro Manila was one of the three regions in the country that had the highest number of DF cases (25,208) [6]. Then, in 2017 it ranked second with the highest increase (19.1%) in DF cases (4706) from 1 January to 3 June (morbidity week 1–22) compared with the same period in 2016 [7]. In spite of many health programs enacted by the government to control vector mosquitoes and manage DF infection, there is still an increase in the incidence of DF. Because DF epidemiology and ecology are strongly associated with human habits and activities [8], assessing knowledge, attitude, and practices (KAP) is deemed necessary, yet, at present, to the best of our knowledge, no study has been done to assess the KAP regarding DF in Metro Manila.

Community-based KAP studies have been done to assess the KAP of different communities in other countries. However, most of them have included only community-based samples and investigation on samples with clinical or serologically-confirmed DF diagnosis remains inadequate. To our knowledge, only two community-based case-control studies have been done. Chen et al. [9] interviewed patients with DF who were randomly chosen from a web-based reporting system through telephone interviews, whereas Kenneson et al. [10] performed clinical ascertainment and community screening to interview households with and without DF infections by identifying acute or recent DF infections. However, these studies had limitations in their data collection methods. The first study limited collection to individuals and households with telephones, which had only a 50% response and completion rate among respondents [9]. The second study collected data among households with acute or recent DF infections, suggesting a self-report bias, as members of these households may have already acquired knowledge and changed their behavior or attitude towards DF during their surveillance [10]. Thus, we tried to address these limitations by performing hospital-based face-to-face interview surveillance among patients with DF through the use of a questionnaire. Although this method has been reported to have good response and acceptance rate (99%) and a low refusal rate (1%) among in- and out-patients [11,12], this also allowed us to capture patients’ knowledge and attitude and their family’s/household’s practices against DF during the onset (acute phase (febrile-critical) of the infection [1]). We assumed that during the onset of DF, they had no acquired knowledge on DF nor had they changed their attitude or behavior toward DF. Moreover, studying this group will provide important benchmark information on identifying and confirming which of the three KAP domains plays a vital role in the presence and spread of disease which, in turn, would help structure more targeted and proactive community-wide disease prevention and control programs.

Previous KAP studies have also reported that sociodemographic data such as income, employment, education, marital status, religion, sex, age, location, socio-economic status, type of residence, and DF history were associated with KAP [13,14,15,16,17,18,19,20,21,22,23]. However, to our knowledge, no study has investigated the association between clinical parameters (e.g., diagnosis, platelet count), clinical symptoms (e.g., fever, rash, abdominal pain), and KAP. Thus, the inclusion of clinical parameters collected during patient hospitalization could give us significant clues as to whether particular attributes are associated with the disease [24].

Several community-based KAP studies have also investigated the correlation among the KAP domains. Harapan et al. [13] reported that good knowledge is positively associated with good practice. This is parallels with the report by Alyousefi et al. [25] that poor knowledge on DF has significant positive association with poor preventive practices. However, other similar studies had different results. Kumaran et al. [26] and Shuaib et al. [14] reported that knowledge on causes, signs, symptoms, mode of transmission, and preventive practices against DF are not correlated with the practice of preventive measures against DF. Aside from these, two case-control studies reported which preventive practices are protective factors against DF. Regression models revealed that removing trash and stagnant water from around the residence, using mosquito repellent oils, use of mosquito bed nets, fumigation inside the house, and piped water inside the house can reduce the risk and vulnerability to DF infection [9,10].

On the basis of the literature presented, we hypothesized that pediatric and adult patients with DF would have lower levels of KAP domains than the pediatric and adult controls, respectively. In line with this, different clinical variables would be significant predictors of KAP among the pediatric and adult patients with DF. Moreover, patients’ knowledge and attitude on DF would not have a significant positive relationship with their practices against DF, compared with that of pediatric and adult controls, which would imply that low practice levels exposed the patients to the infection. Therefore, this study aimed to assess and compare the KAP of pediatric patients with DF and pediatric controls, and adult patients with DF and adult controls. We also sought to identify the predictors of KAP domains by socio-demographic profiles, clinical parameters, and symptoms; analyze the relationship among the KAP domains; and identify protective factors against DF. The results will be used as a springboard in identifying and recommending a structure for more targeted and proactive community-wide DF prevention and control programs.

## 2. Materials and Methods

### 2.1. Study and Sampling Design

This case-control study involved clinically or serologically confirmed patients (pediatrics *n* = 233; adults *n* = 17) and community-based controls (pediatrics *n* = 233; adults *n* = 17). Patients with DF were admitted in three public tertiary (>100 beds) hospitals in Metro Manila, Philippines, at San Lazaro Hospital, a referral facility for infectious/communicable diseases; Quezon City General Hospital; and Pasay City General Hospital, whereas the controls were community (adults)- and school (pediatrics)-based samples. We used the 1:1 ratio (one case patient/one control) with an assumed odds-ratio of ≥2, power (1-β) of 0.80, 0.05 significance level, Z_α_ = 1.96 [27]. Community-based adult controls were compared with adult patients with DF, whereas pediatric patients with DF were compared with school-based grade 3 to grade 12 students (8 to 18 years old). However, we failed to control potential confounders by matching them in terms of age, gender, and grade level because availability and participation rates among the controls were low. Thus, we chose those who were eligible on the basis of the inclusion criteria, and those who were available and willing to participate. The collection was done during the rainy season where high DF transmission occurs from 26 July to 26 November 2017.

### 2.2. Participant Inclusion and Exclusion Criteria

A semi-structured bedside interview was done among pediatric (18 years old and below) and adult in-patients (19 years old and above) with serology-confirmed or clinically diagnosed DF, who were conscious and able to read and write. Excluded were those who were not able to comply with consent procedures, or with life-threatening comorbidities. Controls were sampled individuals who had no signs and clinical symptoms of DF and who had no family member hospitalized for or diagnosed with DF at the time of interview. For more information about the transcript used in the semi-structured interview, see Supplementary File S1.

### 2.3. Ethical Considerations and Data Collection Procedures

The study was conducted on the basis of international and local ethical guidelines: Declaration of Helsinki, International Council for Harmonization-Good Clinical Practice (ICH-GCP) Guidelines, and National Ethical Guidelines for Health Research [28,29,30], and reported on the basis of the Strengthening The Reporting of OBservational Studies in Epidemiology (STROBE) statement checklist (Supplementary File S2). It was reviewed and approved by the Institutional Ethics and Review Boards (IERBs) of each participating hospital: Research Ethics and Review Unit of San Lazaro Hospital, Research Ethics and Technical Committee of Pasay City General Hospital and Planning, Development, and Education and Research office of Quezon City General Hospital (ethics approval numbers: RERU-SLH 2017-016 E and PGH-RETC-01-0-091417001DENGUE).

Informed consent was obtained from all the controls and patients and/or their parent or legally authorized representative (LAR), or caregiver, especially in the case of those who were under 18 years old. They were asked to read and sign an informed consent form (verbal assent for children aged 7–12 years and assent form for children aged 12–15 years) in Filipino before their participation in the study. They were also informed that their participation in the study was voluntary and they may stop their participation any time. Recruitment and interviews of patients were completed by one trained investigator and were supervised by physicians, nurses, and co-investigators assigned at each hospital. The controls were recruited and interviewed by guidance and counseling personnel and a psychologist on the basis of the inclusion and exclusion criteria. To avoid bias, interviews were done with a consistent pre-determined instructions and questions using structured forms and a pre-tested self-report questionnaire. This was done with the expectation of fairly consistent data from one participant to another. Forms and questionnaires of each participant were coded for their protection and privacy.

### 2.4. Forms and Instruments

#### 2.4.1. Explanatory Variables

Socio-demographic profile, clinical parameters, and symptoms: Both patients and controls were asked about their personal information such as age, civil status, gender, educational attainment or employment status, family monthly income, and the DF history of their family and themselves. Patients’ clinical parameters such as admitting diagnosis, serologic test results (non-structural protein 1 antigen (NS1Ag) and BLOT: immunoglobin G antibody (IgG) and immunoglobin M antibody (IgM)) and laboratory data (i.e., complete blood count (CBC) with platelet count) were obtained from medical charts that were used to identify their current DF phase (acute: febrile to critical and recovery phase). Clinical symptoms or chief complaints were also asked.

#### 2.4.2. Response Variables

KAP regarding DF was developed by Shuaib et al. [14] in Jamaica, which was pretested and then completed three Delphi method review rounds for question and response construction and purpose of the questionnaire. The questionnaire has three domains: 29-item knowledge (dengue symptoms, modes of transmission, preventive practices, and disease management), 3-item attitudes (seriousness, risk, and prevention), and 12-item practices (mosquito–human contact and eliminating breeding sites) [14]. Knowledge and attitude domains pertain to each participant’s self-report of knowledge and perception towards DF, whereas the practice domain involves each participant’s household-report of the preventive practices against DF. We added two items in the list of sources of information (e.g., social media and “barangay” or villages and community) and one item in practice (dengue vaccine). A three-point scale, “yes”, “no”, and “I don’t know” was used in the knowledge domain. Correct responses were scored 1, otherwise, scored 0 [20]. A five-point scale, “strongly agree” to “strongly disagree” was used to identify participants’ attitudes where “strongly agree” scored 2 and “agree” scored 1. Likewise, one item in practices (frequency of cleaning ditches and containers with water) used a four-point scale of “always” to “never” where “always”, “often”, and “sometimes” were scored 3, 2, and 1, respectively. Approval was obtained from one of its authors for the use of the test with modifications, forward translation (English to Filipino), expert validation (content, construct, face), back translation (Filipino to English), and pilot testing procedures. The translated questionnaire in Filipino was tested for internal consistency (Cronbach’s alpha), and results evidenced KAP domains’ acceptable internal consistency Cronbach’s alpha of α = 0.75, α = 0.76, and α = 0.76 for the three domains, respectively.

### 2.5. Statistical and Data Analysis

Statistical analysis was done using Statistical Package for Social Sciences (SPSS) version 25 (IBM Corp., Armonk, NY). We compared the groups—pediatric patients and pediatric controls, and adult patients and adult controls—by their mean scores in each KAP domain using independent samples *t*-test. Then, we conducted multiple linear regression analysis where socio-demographic and clinical variables (dummy variables (i.e., 0 or 1) for categorical variables) were inputted in the model using a stepwise method in backward selection to identify significant (*p* < 0.05) predictors of KAP among patients with DF. To calculate the correlation values between the KAP domain scores, Spearman’s rank correlation (r_s_) (two-tailed) and the fisher’s R-to-Z transformation to obtain confidence interval (CI) were used, as Shapiro–Wilk and Kolmogorov–Smirnov normality tests revealed that the scores were not normally distributed [20]. All preventive practices were used in a logistic regression analysis to identify protective factors against DF infection in pediatric and adult samples. All significant factors (*p* < 0.05) were put in the multiple regression analysis using stepwise backward selection method.

## 3. Results

### 3.1. Socio-Demographic Profile, Clinical Parameters, and Symptoms

Initially, 350 patients with DF participated in the study. However, we excluded those who had incomplete responses (*n* = 15, 4.3%) and those whose responses came from a family member instead of the patient themselves (*n* = 85, 24.3%). Thus, data from 500 participants comprising 250 patients with DF (pediatrics *n* = 233 (93.2%); adults *n* = 17 (6.8%)) and 250 controls (youth *n* = 233; adults *n* = 17) were included in the final analysis. The profile of the participants is shown in Table 1. Pediatric patients with DF had a mean (M) age of 13, and an SD (±) of 3.16 years. A total of 56.7% were males, 46.9% were in junior high school, and 84% belonged to a family with a monthly income of ≤10,000 pesos. The age of adult patients ranged from 19 to 49 years old (M, 29.9 ± 10), 64.7% were females; 73.3% were single, 61.5% were employed, 70.6% belonged to a family with a monthly income of ≤10,000 pesos, and 70.6% belonged to a family with ≤5 members. All (100%) adult patients and the majority (77.7%) of pediatric patients with DF had dengue with warning signs. A large proportion of pediatric patients and adult patients had no DF history (92.7% and 93.3%, respectively), had no family DF history (69% and 88.2%, respectively), and were in the acute (febrile-critical) phase of the infection (80.7% and 70.6%, respectively). More than half (68.2%) of pediatric patients and the majority (88.2) of adult patients had thrombocytopenia (9900/mm^3^). Nearly half (43%) of pediatric patients and 35.3% of adult patients had petechiae or rashes.

Furthermore, pediatric controls had a mean age of 14.11 (±1.88) years with almost half (47.6%) belonging to the 14–16 age group, whereas adult controls had a mean age of 26.6 (±6.07) years that ranged from 20 to 46 years old. Half (51.1%) of pediatric controls were males, whereas 64.7% of adult controls were females. All (100%) of youth controls and the majority (94.1%) of adult controls were single. Most of the pediatric (92.7%) and adult controls (93.8%) belonged to a family with a monthly income of ≥10,000 pesos. There was a preponderance of pediatric (86.7%) and adult (94.1%) controls who had no DF history. More than half of pediatric (79.4%) and adult controls (58.8%) had no family DF history.

### 3.2. Mean Score Difference of Knowledge, Attitude, and Practice between Patients and Controls

An independent samples *t*-test revealed that pediatric patients with DF had significantly higher mean scores in knowledge (*p* < 0.001) and attitude (*p* < 0.001) domains than pediatric controls (Table 2). However, pediatric patients with DF obtained significantly lower mean score in practice domain (*p* < 0.05) than pediatric controls. In adult samples, adult patients with DF had significantly lower mean scores in knowledge (*p* < 0.001) and practice (*p* < 0.001) domains than adult controls.

### 3.3. Predictors of Knowledge, Attitude, and Practice

Multiple linear regression analysis found significant regression equations in all KAP domains among pediatric patients with DF, as shown in Table 3. It shows that knowledge increased significantly more in pediatric patients who were in senior high school, whereas it decreased significantly more in pediatric patients who were in college and those who had DF for the first time. Being in senior high school also tended to increase pediatric patients’ attitude. Additionally, the longer they stayed in the hospital, the higher their attitude score became. However, the older their age, their attitude scores lowered. Further, practice scores tended to decrease among those with severe dengue, yet, tended to increase in those pediatric patients who had petechiae or rash. No significant predictors were found among adult patients with DF.

### 3.4. Correlation among Knowledge, Attitude, and Practices

Spearman rank correlation revealed that, although not statistically strong (*r_s_* = 0.2), there was a significant positive correlation between knowledge and attitude domains of pediatric patients with DF, as shown in Table 4. As hypothesized, there was no correlation found in knowledge–practice and attitude–practice domains of both pediatric and adult patients with DF. Among controls, only youth controls had obtained significant positive correlations among the KAP domains, wherein a relatively strong correlation was found between knowledge–practice domains with a correlation coefficient of 0.42 (95% CI: 0.34–0.57).

### 3.5. Protective Factors against DF

All preventive practices were used in a logistic regression analysis to identify protective factors against DF. Then, after a multiple regression analysis, use of mosquito-eating fish, dengue vaccine, use of screen windows, and performing at least one preventive practice against DF were found to be protective factors against DF among youth samples (pediatric patients with DF and pediatric controls), as shown in Table 5. Among adults (adult patients with DF and adult controls), only the use of screen windows was identified as a significant protective factor against DF, with an adjusted odds-ratio (aOR) of 23.9 (95% CI: 2.08–275.2, *p* = 0.01). For both youth and adult samples, mosquito-eating fish, screen windows, and dengue vaccine were identified as protective factors against DF infection. The strongest factor in the model was use of mosquito-eating fish, with an adjusted ratio (aOR) of 8.69 (95% CI: 3.67–20.57, *p* ≤ 0.001).

## 4. Discussion

Pediatric patients with DF had significantly higher mean scores in knowledge and attitude than pediatric controls, who, in turn, as expected, had a significantly higher mean score in the practice domain compared with pediatric patients with DF. As expected, adult patients with DF had significantly lower mean scores in knowledge and practice domains than adult controls. Being in senior high school, having had days in the hospital, and having a rash were predictors of KAP among pediatric patients with DF, whereas no significant predictors were found among adult patients with DF. There was a significant positive correlation between knowledge and attitude (*p* < 0.01) of pediatric patients with DF, however, similar with adult patients with DF, these domains did not correlate with their practices against DF. Moreover, mosquito-eating fish, screen windows, and dengue vaccine were protective factors against DF, although further community-based studies should confirm these results.

Our study extends the investigations done by Chen et al. [9] and Kenneson et al. [10] that focus on clinical and laboratory-confirmed DF infections as the primary outcome of interest rather than preventive practices. As this was a hospital-based study, this allowed us to easily identify individuals with positive cases according to clinical diagnostic criteria and laboratory-confirmation of DF diagnosis. Our study provides the first report on the use of clinical ascertainment through hospital-based surveillance among pediatric and adult patients with DF and the first description on the difference of KAP regarding DF between patients with DF and community-based controls. It also provided the first investigation on the association of clinical data to KAP domains, correlation among the KAP domains and protective factors against DF among patients with DF and community-based controls.

The significantly high mean scores in knowledge and attitude, as well as the low mean scores in practice among pediatric patients with DF compared with their counterparts, may be explained by factors associated with being hospitalized. It may be possible that hospitalization caused pediatric patients to gain more knowledge about DF compared with pediatric controls. Being diagnosed with DF increased their awareness regarding DF, and multiple encounters with different healthcare providers or other patients might have increased their knowledge about DF. Having DF history was also found to be a significant determinant of high knowledge mean scores among pediatric patients with DF. Although only 7.3% had DF before, this may appear obvious; however, past experiences such as infection, which is prevalent among children [29], also increased pediatric patients’ knowledge about DF. Moreover, the significantly high score obtained by pediatric controls in the practice domain implies that they had good practice against DF compared with pediatric patients with DF. This seemed to be also true among adult controls who had higher mean scores in the practice domain than adult patients with DF, which may explain why controls, in general, did not have DF.

Previous studies have reported that there is a significant positive correlation between education and level of knowledge and attitude toward DF [19,23]. However, in our study, pediatric patients who were in senior high school tended to have increased knowledge and attitude on DF compared to those in college or university. One possible reason could be that senior high schools may have included contents about DF in their curriculum that may have increased the knowledge and attitude levels of pediatric patients in senior high school. As the age of pediatric patients with DF increased, their attitude towards DF decreased. Hospitalized younger children were reported to be very conscious about their health and expressed very positive health attitudes compared with older children [31,32]. This may be brought about by the fact that younger children who are hospitalized are more vulnerable to emotional upset and they experience greater anxiety, arising from separation from parents [33,34].

Another significant predictor of attitude towards DF was the number of days in hospital. The longer individuals stayed in hospital, the more they perceived DF as serious and threatening, which may have been due to their experience of anxiety towards medical settings and receiving of medical care [35]. Specifically, they had fear of medical procedures such as injection needles [36] (daily drawing of blood to check their platelet counts) and they perceived medical professionals such as doctors and nurses as inflictors of trauma [37]. Aside from these, hospitalization also increased the chance of children being dissatisfied with their hospital stay situation, due to factors such as food conditions, also experiencing anxiety because of limited physical activities such as being absent in school and having a limited chance of spending time and playing with peers and friends or siblings [38,39].

The appearance of petechiae or rash among pediatric patients may explain, in part, why it was found to be a significant predictor of the practice domain. The presence of petechial rash (which is also described as “isles of white in the sea of red”) and pruritus (severe itching of the skin) occur towards the end of acute (febrile) phase and the beginning of the recovery phase [1]. This could mean that those pediatric patients who were already having rashes during the interview may have already changed their family/household members’ behavior and started performing the preventive practices against DF, thus having higher practice mean scores. Severe dengue was a significant predictor of decreased practice among pediatric patients with DF. Pediatric patients with severe dengue, compared to those who had other DF diagnoses, had the significantly lowest mean score in the practice domain. A total of 50% of pediatric patients with severe dengue had to be confined in the intensive care unit (ICU). They were interviewed only after ICU confinement which was, on average, the fifth day of hospitalization. The time spent in the ICU might have decreased the opportunity of their family/household members to immediately perform the practices against DF, thus having a lower practice mean score. In general, although the regression models obtained low *R*-squared values, it is still important to investigate how the changes in the values of social and clinical variables are associated with the changes in the values of depressive and anxiety symptoms scores which is represented by the significant coefficients.

There was a positive correlation found between knowledge and attitude domains of pediatric patients with DF. Although it was considered a weak association, and relatively lower correlation coefficient compared with a previous study (r_s_ = 0.37) [20], it is still valuable to note that there was a good translation of knowledge to attitude on DF among pediatric patients with DF. Their knowledge on dengue symptoms, modes of transmission, preventive practices against DF, and disease management tended to change their beliefs that DF is a serious and threatening disease. Although pediatric patients’ knowledge correlated with their attitude towards it, as expected, both knowledge and attitude did not correlate with their practices against DF. This clearly signifies that the translation of knowledge and attitude to practice among pediatric patients was poor. This was also found to be true among adult patients with DF. This means that although pediatric and adult patients with DF were knowledgeable about the symptoms of DF, vector breeding site control, and transmission modes of DF, and had the perception of DF as a serious and threatening disease, it did not lead to a change in their behavior of performing the preventive practices against it. This implies that the poor practice against DF might have exposed them to a higher risk of contracting the disease.

Multiple logistic regression analysis on the practices revealed that use of mosquito-eating fish, screen windows, and dengue vaccine were protective factors against DF. Studies have found that larvivorous fish (*Gambusia affinis*), the common guppy (*Poecilia reticulata*), or Cyprinidae or Tilapia spp. can be effectively used to control the mosquito population at their larval stages [40,41,42]. Moreover, in our study, we assumed that the use of screen windows equates to the use of glass windows in air-conditioned rooms among controls, especially the youth. Screen and glass windows could be potential forms of reducing DF transmission by the reduced exposures to vectors that enter homes through open windows [9]. These may not have been available to the patients with DF because the majority (85.2%) of them belong to households with low monthly family income, thus, increasing their vulnerability to DF infection.

Surprisingly, dengue vaccine was found to be a protective factor against DF among youth samples. Another limitation of this study was that we could not rely on the participants’ responses about their dengue vaccine acquisition history. We had no means of confirming whether they were vaccinated or not. Thus, this result may require more intensive community-based studies to identify whether it could truly be a protective factor against DF infection.

We also identified different factors that limited the generalizability of our findings: a small sample size of adult patients with DF, failure to match patients with controls, confounding effect of economic status and hospitalization, and false positive responses. First, limitation was the relatively small sample size of adult patients with DF, which is a possible reason why no significant predictors of KAP were found among this population. The underrepresentation of adult patients may have been caused by the reason that DF is more prevalent in children and a younger age group and only those adult patients who reported severe symptoms and those who needed special attention were admitted to hospital while others were asked to stay at home and consult outpatient clinics. A second limitation was the failure to match the pediatric patients and pediatric controls at least in terms of age, which also limits the generalizability of the findings. As we mentioned previously, matching was difficult because not all the control sample that we approached were available and willing to participate. The third limitation was that participating hospitals were public tertiary hospitals, where most patients belong to low-income families; thus, association of income with the domains was hard to estimate. Fourth, only in-patients were included in this study, limiting the analysis to those admitted to hospitals. Therefore, we recommend that future studies also include out-patients to see whether hospitalization confounds the association between the domains and the DF infection. The inclusion of patients who had DF was also found to be a confounding variable in the level of knowledge and attitude; thus, future studies should consider focusing on patients who had no history of DF. Lastly, our data collected from both patients with DF and controls, through the use of questionnaire, were subjective, which might have produced false positive responses. We had no means of confirming whether the participants, especially the patients, really had mosquito-eating fish at home, if they had been using screen windows, or if they had been vaccinated with dengue vaccine; thus, future studies should include direct household observation to validate these results.

## 5. Conclusions

Pediatric and adult patients with DF admitted in three hospitals in Metro Manila, compared with their counterparts, had lower mean scores in the practice domain, and knowledge and attitude were not correlated with practice, highlighting the importance of behavioral change for knowledge and attitude to have a significant effect on practices against DF. Health programs should focus on translating knowledge and attitudes into more effective practices against DF through behavior change. Many programs continue to focus only on changing people’s knowledge and on raising awareness, rather than physical activity programs, which are more successful at producing behavior change [43]. Thus, we recommend two comprehensive health programs that aid the successful translation of knowledge and attitude to better practice. The Communication for Behavioral Impact (COMBI) is a comprehensive strategy that uses communication of knowledge to have a significant effect upon behavioral change (making people becoming aware, informed, convinced, and deciding to act, then repeating and maintaining that action) or increased practices against DF [26,44]. Moreover, another model that facilitates behavioral change that could increase the translation of attitude to practice among children and adolescents is the Health Belief Model (HBM). This model suggests that a change in behavior can be expected if a person perceives themselves to be at risk or susceptible to the disease (perceived susceptibility), that the disease will have serious consequences (perceived severity), a course of action will minimize consequences (perceived benefits), and the benefits of action will outweigh the cost of barriers (perceived barriers) and self-efficacy [45]. Both models should be used in changing behavior, not only at individual and household levels, but also at the community level, as community participation, including schools and especially among children, is necessary to effectively control the vector mosquitoes [46].

## Figures and Tables

**Table 1:** Socio-demographic profile, clinical parameters, and clinical symptoms among pediatric and adult patients with DF and pediatric and adult controls.

			Patients with DF			Controls		
			Total	PP	AP	Total	PC	AC
			*n* = 250	*n* = 233	*n* = 17	*n* = 250	*n* = 233	*n* = 17
Socio-demographic profile	Gender	Male	138 (55.2)	132 (56.7)	6 (35.3)	125 (50.0)	119 (51.1)	6 (35.3)
		Female	112 (44.8)	101 (43.3)	11 (64.7)	125 (50.0)	114 (48.9)	11 (64.7)
	Age	8–10	54 (21.6)	54 (23.2)		4 (1.60)	4 (1.70)	
		11–13	53 (21.2)	53 (22.7)		93 (37.2)	93 (39.9)	
		14–16	75 (30.0)	75 (32.2)		111 (44.4)	111 (47.6)	
		17–18	51 (20.4)	51 (20.4)		25 (10.0)	25 (10.7)	
		19–21	5 (2.00)		5 (29.4)	3 (1.20)		3 (17.6)
		22–24	2 (0.80)		2 (11.8)	4 (1.60)		4 (23.5)
		25–27	2 (0.80)		2 (11.8)	3 (1.20)		3 (17.6)
		≥28	8 (3.20)		8 (47.1)	7 (2.80)		7 (41.2)
	Education/employment	Grade school	72 (30.1)	72 (31.9)		11 (4.40)	11 (4.70)	
		JHS	106 (44.4)	106 (46.9)		204 (81.6)	204 (87.6)	
		SHS	39 (16.3)	36 (15.9)	3 (23.1)	12 (4.80)	12 (5.20)	
		College	8 (3.30)	7 (3.10)	1 (7.70)	9 (3.60)	6 (2.60)	3 (17.6)
		Employed	13 (5.40)	5 (2.20)	8 (61.5)	10 (4.00)		10 (58.8)
		Unemployed	1 (0.40)	0	1 (7.70)	4 (1.60)		4 (23.5)
	Income (₱)	≤10,000 PHP	192 (83.1)	180 (84.1)	12 (70.6)	18 (7.20)	17 (7.30)	1 (6.30)
		≥10,000 PHP	39 (16.9)	34 (15.9)	5 (29.4)	231 (92.8)	216 (92.7)	15 (93.8)
	Civil status	Single	243 (98.4)	232 (100)	11 (73.3)	249 (99.6)	233 (100)	16 (94.1)
		Married/live-in	4 (1.60)	0 (0.00)	4 (26.7)	1 (0.40)		1 (0.30)
	Household member	≤5 members	128 (55.4)	116 (54.2)	12 (70.6)	174 (69.6)	164 (70.4)	10 (58.8)
		≥6 members	103 (44.6)	98 (45.8)	5 (29.4)	76 (30.4)	69 (29.6)	7 (41.2)
Clinical parameters	Medical diagnosis	DHF w/ws	198 (79.2)	181 (77.7)	17 (100)			
		Severe dengue	6 (2.40)	6 (2.57)				
		Probable	46 (18.4)	46 (19.7)				
	Days in the hospital	≤2 days	170 (76.2)	159 (77.2)	11 (64.7)			
		≥3 days	53 (23.8)	47 (22.8)	6 (35.3)			
	DF history	Had DF	16 (7.30)	15 (7.3)	1 (6.70)	32 (12.8)	31 (13.3)	1 (5.90)
		First-time	204 (92.7)	190 (92.7)	14 (93.3)	218 (87.2)	202 (86.7)	16 (94.1)
	Family DF history	None	155 (70.5)	140 (69.0)	15 (88.2)	195 (78.0)	185 (79.4)	10 (58.8)
		≥1 had DF	65 (29.5)	63 (31.0)	2 (11.8)	55 (22.0)	48 (20.6)	7 (41.2)
	Dengue phase	Acute	200 (80.0)	188 (80.7)	12 (70.6)			
		Recovery	50 (20.0)	45 (19.3)	5 (29.4)			
	Dengue tests	(−) NS1Ag	52 (44.1)	42 (38.9)	10 (100)			
		(+) NS1Ag	66 (55.9)	66 (61.1)				
		(−) IgG	46 (68.7)	41 (67.2)	5 (83.3)			
		(+) IgG	21 (31.3)	20 (32.8)	1 (16.7)			
		(−) IgM	14 (20.9)	13 (21.3)	1 (16.7)			
		(+) IgM	53 (79.1)	48 (78.7)	5 (83.3)			
Clinical Symptoms	Headache	Asymptomatic	206 (82.4)	193 (82.8)	13 (76.5)			
		Symptomatic	44 (17.6)	40 (17.3)	4 (23.5)			
	Fever	Asymptomatic	210 (84.0)	194 (83.3)	16 (94.1)			
		Symptomatic	40 (16.0)	39 (16.9)	1 (5.9)			
	Nausea and vomiting	Asymptomatic	188 (75.2)	177 (76.0)	11 (64.7)			
		Symptomatic	62 (24.8)	56 (24.3)	6 (35.3)			
	Myalgias and arthralgias	Asymptomatic	193 (77.2)	180 (77.3)	13 (76.5)			
		Symptomatic	57 (22.8)	53 (22.9)	4 (23.5)			
	Petechiae (rash)	Asymptomatic	145 (58.0)	134 (57.5)	11 (64.7)			
		Symptomatic	105 (42.5)	99 (43.0)	6 (35.3)			
	Retro-/peri-orbital pain	Asymptomatic	223 (89.2)	208 (89.3)	15 (88.2)			
		Symptomatic	27 (10.8)	25 (7.86)	2 (11.8)			
	Abdominal pain	Asymptomatic	157 (62.8)	145 (62.2)	12 (70.6)			
		Symptomatic	93 (37.2)	88 (37.9)	5 (29.4)			
	Thrombocytopenia	≤9900/mm^3^	72 (28.8)	70 (22.01)	2 (11.8)			
		≥10,000/mm^3^	165 (66.0)	150 (68.2)	15 (88.2)			

DF—dengue fever; PP—pediatric patients; AP—adult patients; PC—pediatric controls; AC—adult controls; JHS—junior high school; SHS—senior high school; ₱—Philippine peso (52.16 USD = 1 ₱); Acute—febrile to critical phase; ws—warning signs; DHF—dengue hemorrhagic fever; (+)—positive; (−)—negative; NS1Ag—non-structural protein 1 antigen; IgG—immunoglobin G antibody; IgM—immunoglobin M antibody.

**Table 2:** Results of independent *t*-test for the difference of knowledge, attitude, and practice (KAP) mean scores between patients and controls.

	Pediatrics			Adults		
	PP (*n* = 233)	PC (*n* = 233)		AP (*n* = 17)	AC (*n* = 17)	
Domains	Mean (SD)	Mean (SD)	*p-*Value	Mean (SD)	Mean (SD)	*p-*Value
Knowledge	19.6 (3.49)	14.5 (6.26)	<0.001	17.7 (3.58)	22.5 (2.69)	<0.001
Attitude	3.46 (1.91)	2.46 (2.12)	<0.001	3.88 (2.23)	4.12 (1.49)	0.72
Practices	9.29 (2.32)	9.83 (2.93)	0.03	7.94 (2.04)	11.1 (1.78)	<0.001

PP—pediatric patients; PC—pediatric controls; AP—adult patients; AC—adult controls; SD—standard deviation.

**Table 3:** Multiple linear regression results showing the predictors of KAP among pediatric patients with DF.

Outcome Variables	Predictors	*R* ^2^	β	*p-*Value
Knowledge	Education (senior high school)	0.09	0.18	0.01
	Education (college)		−0.16	0.02
	No DF history (first-time)		−0.15	0.04
Attitude	Age	0.11	−0.30	0.004
	Education (senior high school)		0.39	<0.001
	Days in the hospital		0.16	0.04
Practice	Severe dengue	0.07	−0.15	0.04
	Rash or petechiae		0.18	0.01

β—standardized beta coefficients; DF—dengue fever.

**Table 4:** Correlation among the KAP domains among patients with DF and controls.

Patients with DF			Controls	
Variables	r_s_ (95% CI)	*p-*Value	r_s_ (95% CI)	*p-*Value
Knowledge-attitude				
Pediatrics	0.20 (0.08, 0.13)	0.002	0.34 (0.24–0.48)	<0.001
Adults	0.02 (−0.51, 0.59)	0.95	−0.05 (−0.50, 0.60)	0.84
Knowledge-practice				
Pediatrics	0.06 (−0.06, 0.20)	0.36	0.42 (0.34–0.57)	<0.001
Adults	−0.39 (−0.81, 0.24)	0.12	0.03 (−0.58, 0.52)	0.91
Attitude-practice				
Pediatrics	−0.04 (−0.15, 0.10)	0.57	0.23 (0.13–0.38)	<0.001
Adults	−0.13 (−0.62, 0.48)	0.62	0.19 (−0.41, 0.68)	0.46

r_s_—Spearman rank correlation coefficients; 95% confidence intervals (CI) were transformed using Fisher’s R-to-Z.

**Table 5:** Multiple logistic regression model of predictors of absence of DF infection.

Likelihood Ratio Estimates						
Practices	DF	β	SE	Wald *X*^2^	aOR (95% CI)	*p*-Value
Pediatrics						
screen windows	1	1.45	0.31	21.15	4.25 (2.29–7.88)	<0.001
eliminate standing water	1	−1.40	0.41	11.96	0.24 (0.11–0.54)	0.001
mosquito-eating fish	1	2.03	0.45	20.70	7.62 (3.18–18.3)	<0.001
does nothing to reduce mosquitoes	1	0.98	0.37	6.87	2.67 (1.28–5.57)	0.009
dengue vaccine	1	1.60	0.28	32.58	4.95 (2.86–8.56)	<0.001
covering water containers	1	−2.32	0.53	19.15	0.10 (0.03–0.28)	<0.001
Adults						
professional pest control	1	1.82	0.93	3.88	6.20 (1.01–38.1)	0.05
screen windows	1	3.17	1.25	6.49	23.9 (2.08–275)	0.01
Both						
screen windows	1	1.53	0.30	25.92	4.60 (2.56–8.28)	<0.001
eliminate standing water	1	−1.41	0.39	13.20	0.24 (0.11–0.52)	<0.001
mosquito-eating fish	1	2.16	0.44	24.23	8.69 (3.67–20.6)	<0.001
does nothing to reduce mosquitoes	1	0.70	0.36	3.83	2.01 (1.00–4.04)	0.05
dengue vaccine	1	1.49	0.27	30.32	4.42 (2.61–7.55)	<0.001
covered water containers	1	−2.01	0.49	17.09	0.13 (0.05–0.35)	<0.001

DF—degree of freedom; β—standardized beta coefficients; SE—standard error; Wald *X*^2^—Wald chi-square; aOR (95% CI)—adjusted odds-ratio 95% confidence interval.

## Supplementary Materials

The following are available online at https://www.mdpi.com/1660-4601/16/23/4705/s1, Supplementary File S1: Semi-structured interview transcript; Supplementary File S2: STROBE Statement.
