# Supplementary material for: Knowledge, Attitude, and Practices Regarding Dengue Fever among Pediatric and Adult In-Patients in Metro Manila, Philippines
Source: Int J Environ Res Public Health. 2019 Nov 26;16(23):4705. doi: 10.3390/ijerph16234705 (PMC6926575; doi:10.3390/ijerph16234705)
Supplement: Supplementary file 1 [file ijerph-16-04705-s001.pdf]

Supplementary File 1

Semi-structured interview transcript

Please follow the instructions and the script.

I. Informed consent

Interviewer:

1. Hi! Good morning. I am/My name is______________________. I'm a researcher studying the knowledge, attitudes and practices regarding dengue fever among patients with dengue.

2. I'd like to invite you/your child to join our study. Your/your child's participation in this study is entirely voluntary and you can leave or stop any time you want. This will only take 10 to 15 minutes of your time.

3. If you agree to join, I will just ask you some questions about you and your family, then I will give you a survey questionnaire about knowledge, attitude and practices regarding dengue for you to answer.

-Pause and let the patient and/or parent decide to join or not.

-If yes, ask about the patient's age.

Interviewer:

4. Before we start, can I ask your age, please?

NOTE: verbal assent: ages 7-12 years with informed consent of parent, LAR or caregiver
assent form: ages 12-15 years
informed consent: age 15 and above (for patients under 18 years old, parents, LAR or caregiver co-sign the informed consent)

-Prepare the informed consent and/or assent form (two copies each)

NOTE: Please follow the script in the Informed consent or Assent form (highlight the foreseeable **risk, incentives, benefits and confidentiality management, and contact numbers for grievance and complaints**).

-Once finished, ask the patient, parent, LAR or guardian if they have questions, then address them.

-Then, ask them to write their name, affix their signature and write the date in the informed consent or assent form in their appropriate sections.

-If the patient cannot read or write, ask for a witness to sign.

-After they've signed, write your name and affix your signature and date today.

II. Profile sheet: Don't force the patient to answer all the questions.

-Write the hospital, room and bed number, attending physician, and date of interview in the Profile sheet.

5. When is your birthday?

6. Are you single or married? (only for adult patients)

- encircle his/her gender.

7. Are you studying? (If yes, proceed to #9; if no, proceed to number #11)

8. What is your grade level? (proceed to # 10)

9. What is the name of your school/company? Where is it located?

10. Are you working? (If yes, go back to #10)

11. Are you living with your family? (If no, ask probing questions if necessary)

12. How many are you in your family?

13. How much is your family's monthly income? (for children, ask their parents, LAR or guardian) (Probe by letting him/her choose among the options: below 10,000 pesos, etc.)

14. May I ask about your diagnosis? (What was your doctor's diagnosis?)

15. When were you admitted here?

16. How long have you been admitted here in the hospital?

17. Is this your first time to have dengue? (If no, probe by asking date, diagnosis and frequency)

18. Is there someone in your family who has dengue now? (If yes, probe by asking hospital information [whether hospitalized or not], admission diagnosis, relationship with him/her, etc.)

19. Is there someone in your family who had dengue before? (If yes, probe by asking hospital information [whether hospitalized or not], admission diagnosis, relationship with him/her, etc.)

20. How do you feel right now? Do you feel feverish? or cold? (if yes, ask for temperature)

21. Do you feel any pain anywhere in your body? (probe by asking each symptom and ask the severity of pain)

NOTE: (**Mild (or Grade 1)**: Transient or mild symptoms; no limitation in activity; no intervention required;]**Moderate (or Grade 2)**: Symptom results in mild to moderate limitation in activity; no or minimal intervention required; **Severe (or Grade 3)**: Symptom results in significant limitation in activity; medical intervention may be required.)

22. What is your recent platelet count? (check the medical charts for confirmation)

23. What medicines do you take? (probe by asking treatment interventions like antibiotics, blood transfusion, etc.It may also give clue to other diagnosis)

III. KAP Questionnaire: Please follow this script.

Interviewer:

24. Here is the questionnaire about your knowledge, attitude and practices regarding dengue fever.

25. This questionnaire has 3 parts: 29-item knowledge (dengue symptoms, modes of transmission, preventive practices and disease management), 3-item attitudes (seriousness, risk and prevention) and 12-item practices (mosquito-man contact and eliminating breeding sites).

26. Please put your answer by checking in the appropriate column. Some parts are "yes" or no questions, if you don't know the answer, check I don't know column.

27. Other parts are in a scale of 1 to 5 (Strongly agree, agree, not sure, disagree, strongly disagree) and scale of 1 to 4 (Never, sometimes, always or never).

28. Do you have questions? (If none, please tell the patient to start)

29. If you have questions about the items, please don't hesitate to ask me.

NOTE: If the patient cannot write, ask his/her parent, guardian or LAR to write (only) his/her answers.

-Once finished, check if all items were answered. If there are missed items, ask the patients to answer it.

-Give the dengue pamphlet to the patient and discuss the information in it.

-Ask the patient if they want to receive a copy of the output of this study. If yes, ask for their email address.

- Give a copy of the informed consent to the patient.

-Lastly, give incentives to the patient.

30. Thank you for joining in our study!

NOTE: Do not leave any form with the patient's identifiable information

Supplementary File 2

STROBE Statement—checklist of items that should be included in reports of observational studies

	Item No	Recommendation	Subheading of article
**Title and abstract**	1	(*a*) Indicate the study's design with a commonly used term in the title or the abstract<br>*The design, case-control study was mentioned in the abstract*	*Abstract*
		(*b*) Provide in the abstract an informative and balanced summary of what was done and what was found<br>*A structured abstract that includes background, method, results and conclusion parts which contain balanced and informative summary*	*Abstract*
**Introduction**			
Background/rationale	2	Explain the scientific background and rationale for the investigation being reported<br>*We explained in the background that assessing knowledge, attitude and practices (KAP) is deemed necessary, yet, at present, to the best of our knowledge, no study has been done to assess the KAP regarding DF in Metro Manila. We also stated that most of KAP studies have included only community-based samples and investigation on samples with clinical or serologically-confirmed DF diagnosis remains inadequate. To our knowledge, only two community-based case-control studies have been done, however, the methods had limitations, collection procedure and self-report bias. Thus, we tried to address these limitations by doing a hospital-based face-to-face interview surveillance among patients with DF through the use of a questionnaire. This would also allow us to capture patients' knowledge and attitude and their family's/household's practices against DF during the onset of the infection. We assumed that during this time, they haven't acquired knowledge on DF and changed their attitude or behaviour toward DF. Moreover, studying this group will provide important benchmark information on identifying and confirming which of the three KAP domains plays a vital role in the presence and spread of disease which, in turn, would help structure more targeted and proactive community-wide disease prevention and control programs.*	*Background*
Objectives	3	State specific objectives, including any prespecified hypotheses<br>*This study aimed to assess and compare the KAP of patients with DF and controls, identify the predictors of KAP domains, analyse the relationship among them, and identify protective factors against DF. The results will be used as springboard in identifying and recommending structure for more targeted and proactive community-wide DF prevention and control programs.*<br>*We hypothesized that patients with DF would have lower levels of KAP than the controls. Clinical variables would be significant predictors of KAP among the paediatric and adult patients with DF. We also hypothesized that patients' knowledge and attitude on DF would not have significant positive relationship with their practices against DF, compared with that of the controls which would imply that low practice levels exposed the patients to the infection.*	*Background*
**Methods**			

Study design	4	Present key elements of study design early in the paper<br>*We mentioned "This case-control study involved clinically or serologically confirmed patients (paediatrics n = 233; adults n = 17) and community-based controls (paediatrics n = 233; adults n = 17)" in the first sentence of the study and sampling design.*	*Study and Sampling Design*
Setting	5	Describe the setting, locations, and relevant dates, including periods of recruitment, exposure (N/A), follow-up (N/A), and data collection<br>*Patients with DF were admitted in 3 public tertiary (>100 beds) hospitals in Metro Manila, Philippines: San Lazaro Hospital, a referral facility for Infectious/ Communicable Diseases, Quezon City General Hospital and; Pasay City General Hospital while the controls were community (adults) and school (paediatrics) based samples. Community-based adult controls were compared with adult patients with DF while paediatric patients with DF were compared with school-based Grade 3 to Grade 12 students (8 to 18 years old). The collection was done during the rainy season from 26th July to 26th November 2017 in Metro Manila, Philippines.*	*Study and Sampling Design*
Participant s	6	(*a*) *Cohort study*—Give the eligibility criteria, and the sources and methods of selection of participants. Describe methods of follow-up<br>*Case-control study*—Give the eligibility criteria, and the sources and methods of case ascertainment and control selection. Give the rationale for the choice of cases and controls<br>*Paediatric (18 years old and below) and adult in-patients (19 years old and above) had serology-confirmed or clinically diagnosed DF, who were conscious and able to read and write. Excluded were those who were not able to comply with consent procedures, or with life-threatening comorbidities. Controls were sampled individuals who had no signs and clinical symptoms of DF and who had no family member hospitalized for or diagnosed with DF at the time of interview.*<br>*Cross-sectional study*—Give the eligibility criteria, and the sources and methods of selection of participants	*Participant Inclusion and Exclusion Criteria*
		(*b*) *Cohort study*—For matched studies, give matching criteria and number of exposed and unexposed<br>*Case-control study*—For matched studies, give matching criteria and the number of controls per case<br>*We used the 1:1 ratio (one case patient/ one control) with an assumed odds-ratio of ≥2, power (1-β) of 0.80, 0.05 significance level, Z_{α}=1.96. Community-based adult controls were compared with adult patients with DF while paediatric patients with DF were compared with school-based Grade 3 to Grade 12 students (8 to 18 years old). However, we failed to control potential confounders by matching them in terms of age, gender, grade level because availability and participation rates among the controls were low.*	*Study and Sampling Design*
Variables	7	Clearly define all outcomes, exposures (N/A), predictors, potential confounders (N/A), and effect modifiers(N/A). Give diagnostic criteria, if applicable<br>*Outcome or Response variables are: knowledge (dengue symptoms, modes of transmission, preventive practices and disease management), attitudes (perceived seriousness, risk and prevention) and practices (mosquito-man contact and eliminating breeding sites). Explanatory variables or predictors: age, civil status, gender, educational attainment or employment status, family monthly income and family, self DF history, admitting diagnosis, serologic test results (NS1Ag and BLOT: IgG and IgM), platelet count, DF phase (acute: febrile to critical and recovery phase) and clinical symptoms.*	*Explanatory variables and Response variables*
Data sources/	8*	For each variable of interest, give sources of data and details of methods of assessment (measurement). Describe comparability of assessment methods if there is more than one group	*Ethical considerations and data collection procedures*

measureme nt		*We used consistent pre-determined instructions and questions using structured forms and pre-tested self-report questionnaire for both the patients and controls.*	
Bias	9	Describe any efforts to address potential sources of bias<br>*To avoid bias, interviews were done with a consistent pre-determined instructions and questions using structured forms and pre-tested self-report questionnaire. This was done to expect a fairly consistent data from one participant to another.*	*Ethical considerations and data collection procedures*
Study size	10	Explain how the study size was arrived at<br>*All the patients in the three hospitals during the data collection period (July to November) were recruited in the study based from the inclusion criteria.*	
Quantitativ e variables	11	Explain how quantitative variables were handled in the analyses. If applicable, describe which groupings were chosen and why<br>*Explanatory variables or predictors were divided into three categories: socio-demographic profile, clinical parameters and clinical data. Each of the variable was divided into categories. For linear regression analysis, the categorical variables were transformed to dummy variables [i.e., 0 or 1]to identify the predictors of KAP domains which are continuous data. Outcome or Response variables, knowledge, attitude and practice were measured by mean score.*	*Statistical and Data Analysis*
Statistical methods	12	(*a*) Describe all statistical methods, including those used to control for confounding<br>*Multiple linear regression analysis was done by inputting all explanatory variables in the model using a stepwise method in backward selection to identify significant ($P < 0.05$) predictors of KAP among patients with DF.*	*Statistical and Data Analysis*
		(*b*) Describe any methods used to examine subgroups and interactions<br>*We compared the groups: paediatric patients and paediatric controls, and adult patients and adult controls by their mean scores in each KAP domain using independent samples t-test.*	*Statistical and Data Analysis*
		(*c*) Explain how missing data were addressed<br>*Data from participants with incomplete or missing responses were not included in the final analysis.*	*Statistical and Data Analysis*
		(*d*) *Cohort study*—If applicable, explain how loss to follow-up was addressed<br>*Case-control study*—If applicable, explain how matching of cases and controls was addressed<br>*We matched the patients with controls according to the total number of patients in the two age-groups (pediatric and adults) but we failed to match them by the frequency in each age category, gender, grade level, etc.*<br>*Cross-sectional study*—If applicable, describe analytical methods taking account of sampling strategy	*Study and Sampling Design*
		(*e*) Describe any sensitivity analyses<br>*We did not do any sensitivity analyses in this study. However, we did expert validation and measured the internal consistency of the KAP domains using Cronbach's alpha.*	

Results

Participants	13*	(a) Report numbers of individuals at each stage of study—eg numbers potentially eligible, examined for eligibility, confirmed eligible, included in the study, completing follow-up, and analysed	*Socio-demographic Profile, clinical parameters and symptoms*

		Initially, there were 350 patients with DF participated in the study. However, we have excluded those who had incomplete responses (n = 15, 4.3%) and those whose responses came from a family member instead of the patient himself (n = 85, 24.3%).	
		(b) Give reasons for non-participation at each stage<br>*We have excluded those who had incomplete responses and those whose responses came from a family member instead of the patient himself*	*Socio-demographic Profile, clinical parameters and symptoms*
		(c) Consider use of a flow diagram	
Descriptive data	14*	(a) Give characteristics of study participants (eg demographic, clinical, social) and information on exposures and potential confounders<br>*Please see Table 1. Socio-demographic profile, clinical parameters and clinical symptoms among paediatric and adult patients with DF and paediatric and adult controls*	*Socio-demographic Profile, clinical parameters and symptoms*
		(b) Indicate number of participants with missing data for each variable of interest<br>*We included participants who provided complete responses. We have the same number of participants in all response or outcome variables.*	*Socio-demographic Profile, clinical parameters and symptoms*
		(c) *Cohort study*—Summarise follow-up time (eg, average and total amount)	
Outcome data	15*	*Cohort study*—Report numbers of outcome events or summary measures over time	
		Case-control study—Report numbers in each exposure category, or summary measures of exposure<br>*We just reported the mean scores obtained by each participant subgroup in all the KAP domains. Please see Table 2 Results of independent t-test for the difference of KAP mean scores between patients and controls*	*Mean score difference of knowledge, attitude and practice between patients and controls*
		Cross-sectional study—Report numbers of outcome events or summary measures	
Main results	16	(*a*) Give unadjusted estimates and, if applicable, confounder-adjusted estimates and their precision (eg, 95% confidence interval). Make clear which confounders were adjusted for and why they were included<br>*We did not include unadjusted estimates and confounder-adjusted estimates with precision in our study.*	
		(*b*) Report category boundaries when continuous variables were categorized<br>*We did not categorize the outcome variables.*	
		(*c*) If relevant, consider translating estimates of relative risk into absolute risk for a meaningful time period<br>*We did not estimate relative risk or absolute risk in the study.*	
Other analyses	17	Report other analyses done—eg analyses of subgroups and interactions, and sensitivity analyses<br>*Multiple linear regression analysis found significant regression equations in all KAP domains among paediatric patients with DF*<br>*Spearman rank correlation to measure the correlation values among the KAP domains*<br>*All preventive practices were used in a logistic regression analysis to identify protective factors against DF*	*Predictors of knowledge, attitude and practice*<br>*Correlation among knowledge, attitudes and practices*<br>*Protective factors against DF*

**Discussion**			
Key results	18	Summarise key results with reference to study objectives<br>*The first paragraph of the discussion summarizes the key results*	*Discussion*
Limitations	19	Discuss limitations of the study, taking into account sources of potential bias or imprecision. Discuss both direction and magnitude of any potential bias<br>*We mentioned the limitations in the last paragraph of the discussion. We mentioned small sample size of adult patients with DF; failure to match patients with controls; confounding effect of economic status and hospitalization, and; false positive responses as threats to external validity of the study findings.*	*Discussion*
Interpretation	20	Give a cautious overall interpretation of results considering objectives, limitations, multiplicity of analyses, results from similar studies, and other relevant evidence<br>These are our interpretation sin the key results of our study:<br>*1. Being diagnosed with DF, increased their awareness regarding DF and multiple encounters with different health care providers or other patients might have increased their knowledge about DF.*<br>*2. The significantly high score obtained by controls in practice domain implies that they had good practice against DF compared with patients with DF which may explain why controls, in general, did not have DF.*<br>*3. Both knowledge and attitude did not correlate with the practices against DF of patients with DF which clearly signifies that the translation of knowledge and attitude to practice among patients was poor and this might have exposed them to higher risk of contracting the disease.*	*Discussion*
Generalisability	21	Discuss the generalisability (external validity) of the study results<br>*Some generalisabilities of the results from this study were discussed especially in the larger context like patients with DF in general, pediatric patients with DF.*	*Discussion*
**Other information**			
Funding	22	Give the source of funding and the role of the funders for the present study and, if applicable, for the original study on which the present article is based<br><br>This study was supported by the Japan Society for the Promotion of Science (JSPS) Grant-in-Aid for Scientific Research (17H01624, 19H01144), JSPS Core-to-Core Program B. Asia-Africa Science Platforms, and Endowed Chair Program of the Sumitomo Electric Industries Group Corporate Social Responsibility Foundation, which had no role in the design, data collection, statistical analysis, and writing of this manuscript.	*Funding*

*Give information separately for cases and controls in case-control studies and, if applicable, for exposed and unexposed groups in cohort and cross-sectional studies.

Note: An Explanation and Elaboration article discusses each checklist item and gives methodological background and published examples of transparent reporting. The STROBE checklist is best used in conjunction with this article (freely available on the Web sites of PLoS Medicine at http://www.plosmedicine.org/, Annals of Internal Medicine at http://www.annals.org/, and Epidemiology at http://www.epidem.com/). Information on the STROBE Initiative is available at www.strobe-statement.org.
